# Toxicology and teratology of the active ingredients of professional therapy MuscleCare products during pregnancy and lactation: a systematic review

**DOI:** 10.1186/s12906-015-0585-8

**Published:** 2015-03-05

**Authors:** Abdulaziz MS Alsaad, Colleen Fox, Gideon Koren

**Affiliations:** Motherisk Program, Division of Clinical Pharmacology and Toxicology, Department of Pediatrics, Hospital for Sick Children and University of Toronto, 555 University Ave, Toronto, ON M5G 1X8 Canada; Leslie Dan Faculty of Pharmacy, University of Toronto, 144 College street, Toronto, ON M5S 3M2 Canada; Department of Pharmacology & Toxicology, College of Pharmacy, King Saud University, Riyadh, Saudi Arabia

**Keywords:** MuscleCare, Pregnancy, Lactation, Teratogenicity, Safety

## Abstract

**Background:**

The rates of muscle aches, sprains, and inflammation are significantly increased during pregnancy. However, women are afraid to use systemic analgesics due to perceptions of fetal risks. Thus, topical products are important alternatives to consider for those women. Of interest, Professional Therapy MuscleCare (PTMC) has shown to be effective in alleviating the myofascial pain as reported in a randomized, placebo-controlled double-blinded comparative clinical study of five topical analgesics. However, to date, there is no complete review or long-term safety studies on the safety of these products during pregnancy and lactation. Thus, the aim of this article was to review toxicological, developmental, and reproductive effects associated with the use of PTMC products.

**Methods:**

We performed a systematic review on safety of PTMC from all toxicological articles investigating the effects of PTMC’s ingredients. This search was conducted through medical and toxicological databases including, Web of Science, EMBASE, Medline, and Micromedix. Both reported and theoretical adverse effects were extensively reviewed.

**Results:**

Of the 1500 publications reviewed, 100 papers were retrieved and included in the review. Although some ingredients in PTMC products might cause adverse reproductive effects at high systemic doses, these doses are hundreds to thousands fold greater than those systemically available from topical use at the recommended maximum dose (i.e. 10 g/day).

**Conclusions:**

This study provides evidence that, when used as indicated, PTMC is apparently safe for pregnant women and their unborn babies as well as for breastfed infants.

## Background

The rates of muscle aches, sprains, and inflammation are significantly increased during pregnancy, often resulting in lower back and neck pain [1]. These pain symptoms tend to increase as pregnancy advances through the first, second, and third trimesters [1-4]. It has been estimated that approximately two-thirds of pregnant women in the United States experience lower back pain, and more than 50% of them receive little or no treatment from their physicians [4,5]. This could be attributed to the fears of fetal risks associated with the use of systemically administered agents such as non-steroidal anti-inflammatory drugs. Therefore, the use of topical analgesics seems attractive for women afraid of adverse effects of oral medications.

Among topical analgesics, the Professional Therapy MuscleCare (PTMC) has been shown to be effective in alleviating muscle pain in a randomized, placebo-controlled double-blind study of five topical analgesics [6]. The line of PTMC products includes an ointment and Roll-on gel containing ingredients that are topically applied to alleviate the pain caused by inflammation, muscle strain/spasm, and arthritis during pregnancy. To date, the safety of PTMC ingredients during pregnancy and lactation has not been reviewed. Thus, the aim of the current article was to review the available evidence related to fetal safety of PTMC ingredients and to create an evidence-based framework for PTMC use during pregnancy and lactation.

### Physiological changes during pregnancy and the need for topical analgesics

Pregnancy is associated with continuous physiological changes that lead to higher rates of lower back and neck pains [2-4,7,8]. These changes include weight gain, hormonal changes, and muscle separation in the area of the sacroiliac joint. While the hormone relaxin usually relaxes ligaments in the pelvic area allowing the birth process to take place [9], this hormone also relaxes ligaments supporting the spine, often leading to severe pain during pregnancy. This situation is exacerbated by separation of muscles secondary to the increased size of the uterine. The emotional stress might also contribute to back spasms in pregnancy [10]. Pain increases as a result of weight gain burdening the muscles from the neck down, often leading to stiffness. Taking into consideration the large number of pregnant women refusing the use of systemic analgesics, there is an urgent need to evaluate the safety of topical analgesics such as PTMC [4,5].

### Route of exposure and topical use of PTMC

In this article, we retrieved all existing toxicological information regarding PTMC ingredients in the context of the concentrations of each ingredient within these products. Where available, an attempt has been made to focus on studies employing topical rather than oral exposure, and to highlight studies carried out on humans rather than animals or *in vitro* cell culture models. However, since PTMC products have not been directly studied *in vitro* or *in vivo*, their toxicities need to be extrapolated from studies examining PTMC’s ingredients either alone or in studies using similar ingredients to those found in PTMC products. It is important to bear in mind that compounds in similar creams may be presented at different ratios, which might influence the properties of these products. Thus, when examining each ingredient, it is important to remember that these ingredients were invariably studied at different concentrations in animal studies and, thus, extrapolation of data from other creams to PTMC must be done cautiously. Taking into consideration that the principal source of human exposure to PTMC products is through the skin, these products are strictly intended for external use at recommended maximal dose of 10 g/day. Table 1 summarizes the amounts received by a 70 kg person applying 10 g of PTMC.

**Table 1:** **Amounts received by a 70 kg person applying 10 g of PTMC ointment or roll-on gel**

**Active ingredient**	**Amount received from ointment**		**Amount received from roll-on gel**	
	**mg**	**mg/kg**	**mg**	**mg/kg**
MSM	500	7.14	50	0.71
Camphor	300	4.28	300	4.28
Menthol	400	5.71	1000	14.28
Methyl salicylate	100	1.43	--	--
Glucosamine sulfate	300	4.28	20	0.28
Sodium chondroitin sulfate	10	0.14	10	0.14
Eucalyptus Oil	50	0.71	300	4.28
Grape seed oil	100	1.43	--	--
Vitamin E	100	1.43	--	--
Thymol	20	0.28	--	--
Sea cucumber extract	100	1.43	--	--
Aloe barbadensis leaf juice	10	0.14	--	--
Peppermint oil	--	--	300	4.28
Boswellia	--	--	70	1.0
Ilex	--	--	70	1.0
Magnesium	20	0.28	10	0.14

## Methods

### Search method

**Figure 1:**
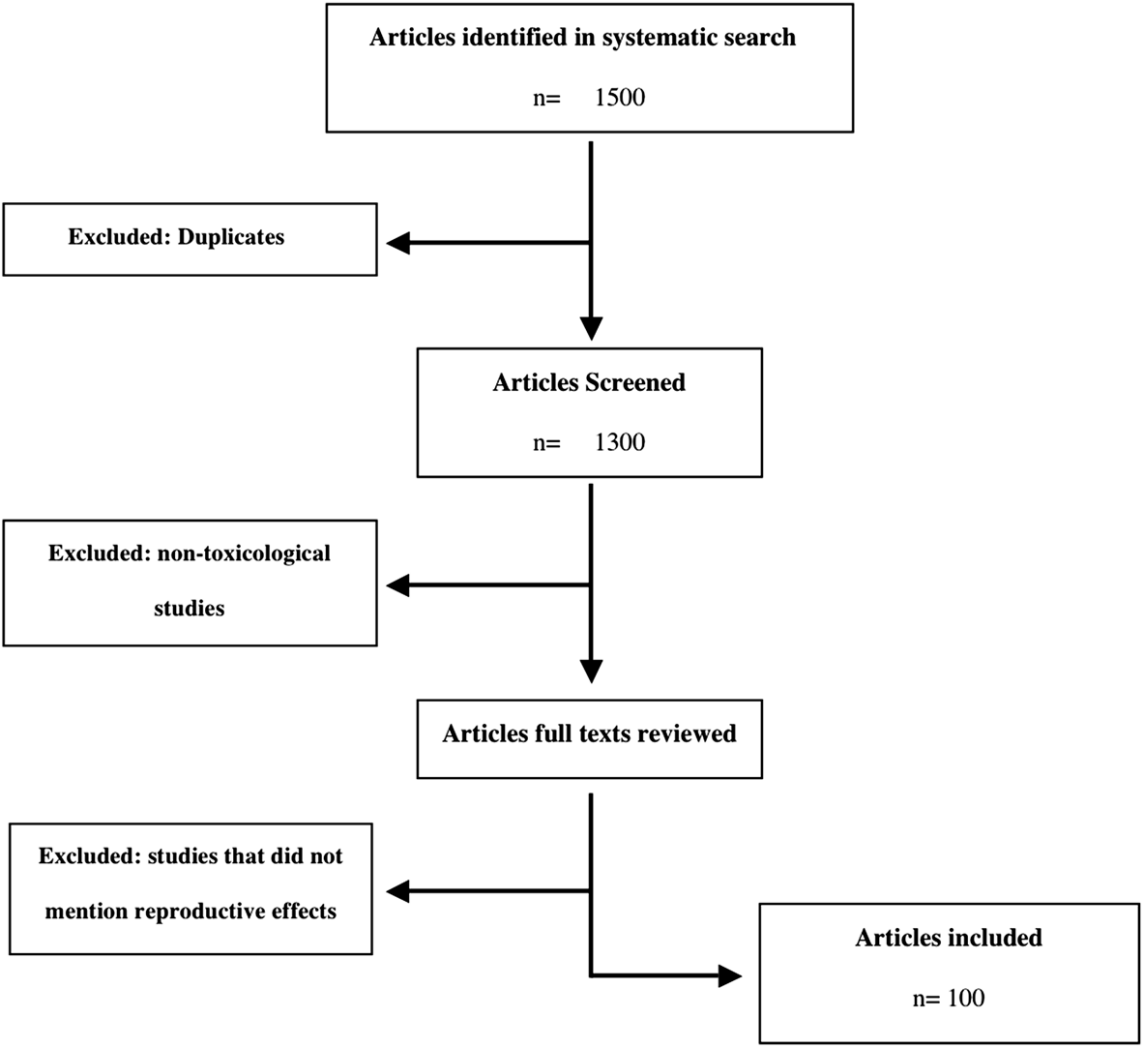
**Study flow chart.**

A professional librarian with expertise in the areas of systematic reviews carried out literature searches using Web of Science, EMBASE, Medline, and Micromedix. The terms used in systematic search were the names of PTMC ingredients and “reproductive effect” with no language restrictions. Studies were selected based reporting toxicological, developmental, and reproductive effects of PTMC ingredients. We excluded all studies that did not mention safety of these ingredients. Of the 1500 publications reviewed, 100 papers were retrieved and included in the review (Figure 1 Study flowchart).

## Results and discussion

### Toxicological, developmental, and reproductive effects of PTMC

The PTMC ingredients are mainly classified as anti-inflammatory, analgesics, circulation enhancers, or tissue repair ingredients (Tables 2 and 3). These ingredients were reviewed with respect to general, developmental, and reproductive adverse effects reported in human and/or animal studies (Table 4 summarizes these adverse effects of ingredients of PTMC).

**Table 2:** **Active ingredients of PTMC ointment and roll-on gel**

**Active ingredients**	**Purpose**	**Content % (Ointment)**	**Content % (Roll-on)**
**MSM** (methylsulfonylmethane)	Topical: anti-inflammatory, Oral: relieves osteoarthritis pain	5.0%	0.50%
**Menthol**	Topical: analgesic and anesthetic	4.0%	10.0%
**Camphor**	Topical: analgesic	3.0%	3.0%
**Glucosamine**	Topical: analgesic, Oral: relieves joint pain, protects against cartilage deterioration	3.0%	0.20%
**Vitamin E**	Anti-oxidant protection of skin	1.0%	Not in roll-on
**Sea Cucumber**	Tissue repair, antioxidant	1.0%	Not in roll-on
**Oil of Wintergreen**	Topical: analgesic, anti-inflammatory	1.0%	Not in roll-on
**Eucalyptus Leaf Oil**	Topical: analgesic, counter-irritant, skin penetration	0.5%	3.0%
**Grape Seed Oil**	Healing damaged tissue	1.0%	Not in roll-on
**Aloe Barbadensis Leaf**	Topical: analgesic	0.1%	Not in roll-on
**Thymol**	Topical: analgesic, anesthetic	0.2%	Not in roll-on
**Chondroitin**	Skin conditioning	0.1%	0.1%
**Peppermint Oil**	Topical: analgesic	Not in ointment	3.0%
**Boswellia**	Topical: anti-inflammatory	Not in ointment	0.7%
**Ilex**	Skin conditioning	Not in ointment	0.7%
**Magnesium chloride**	Viscosity controlling	0.2%	0.1%

**Table 3:** **Inactive ingredients of muscle care ointment and roll-on gel**

**Inactive ingredients**	**Purpose**	**Content % (Ointment)**	**Content % (Roll-on)**
**Cetyl alcohol**	Viscosity increasing	6.0%	Not in roll-on
**Stearyl alcohol**	Emollient, thickening agent	4.0%	Not in roll-on
**Glycerin**	Reduces irritation, prevents dehydration	4.0%	0.6%
**Polysorbate-20**	Surfactant	1.0%	Not in roll-on
**Urea**	Hydrating, penetration enhancer	1.0%	Not in roll-on
**Water**	Hydrating, penetration enhancer	55.1%	8.90%
**Sepigel 305**	Increases viscosity, stability, and shine	2.0%	Not in roll-on
**Optiphen Plus**	Preservative (for paraben-free use)	0.8%	Not in roll-on
**Acrylates Copolymer**	Binder/film former	Not in ointment	9.0%
**Propylene Glycol**	Skin conditioning, penetration enhancer	Not in ointment	3.0%
**Denatured alcohol**	Astringent, masking	Not in ointment	56.25%
**Triethanolamine**	Emulsifying agent	Not in ointment	0.9%

**Table 4:** **Summary of general and reproductive effects of PTMC products**

**Ingredient**	**Species**	**General toxicology**	**Reproductive effects**	**References**
Methylsulfonylmethane	Human	• Topically, there are no adverse reactions.	• Till date, no data.	[11-16]
		• There are no studies on long-term use.		
	Animal	• Orally, it has low toxicity.	• Do not induce structural or fetal anomalies.	
		• Long-term use did not cause adverse events.		
**Camphor**	Human	• Topically, poisonings were reported in children and adults.	• Topically, the frequency of birth defects was less.	[17-28]
		• Orally, it caused fatal symptoms in children.	• Orally, cross the human placenta, however, till date, there are no adverse fetal effects.	
	Animal	• The oral LD_50_ = 1.3 g/kg in mice.	• Orally, there are no congenital abnormalities in rats and rabbit.	
**Menthol**	Human	• Topically, it is safe.	• Till date, no data.	[29-31]
	Animal	• Topically, acute dermal toxicity was reported with LD_50_ = 5 g/kg in rabbit.	• There is no teratogenic effect in mice, rats, hamsters, or rabbits.	
		• Orally, toxicity was reported at LD_50_ = 2.9 g/kg and 3.1 g/kg in rat and mice, respectively.		
**Wintergreen Oil**	Human	• In cosmetics, methyl salicylate is safe, however, might cause local necrosis.	• Till date, no data.	[32-42]
		• There are some reports for tinnitus, diplopia, shortness of breath, and respiratory alkalosis.		
	Animal	• Topically, sub-chronic exposure might lead to kidney damage in rats.	• It is associated with increased risk of abnormalities.	
**Glucosamine Sulfate**	Human	• Topically, it did not cause toxicity or adverse effects.	• There is no increase in risk of malformations.	[43-47]
	Animal	• Topically, it is considered safe.	• Teratogenic effects were not reported in mice or rabbits exposed to glucosamine.	
**Sodium Chondroitin Sulfate**	Human	• Topically, there are no adverse events.	• Till date, no data	[43,48-52]
		• It interferes with progression of osteoarthritis.		
	Animal	• The oral LD_50_ for mice is greater than 10 g/kg.	• There is increased risk of cleft palate and tail abnormalities in mice.	
			• Orally, there are no adverse effects in mice and rabbits.	
**Eucalyptus Leaf Oil)**	Human	• Topically, it is safe.	• Till date, no data.	[53-56]
		• A report of fever and seizure-like motor activity -in slurred speech, ataxia, and muscle weakness were reported in children.		
		• Orally, there are minor side effects.		
	Animal	The oral LD_50_ = 2.5 g/kg in rats.	• There are no adverse outcomes in mice.	
**Grape Seed Oil**	Human	• Topically, it is safe.	• Till date, no data.	[57-60]
	Animal	• There are no safety issues associated with acute and chronic safety studies rats	• The grape seed extract was non-mutagenic in mice.	
			• Several reports showed that LD_50_ for dermal application is greater than 2 g/kg in rats.	
**Vitamine E**	Human	• Topically, it is safe.	• There are no risks of stillbirth, perinatal death, preterm birth, intrauterine growth restriction, or mean birth weight.	[61-66]
			• High doses were associated with reduction in birth weight.	
	Animal	• Topically, it is safe.	• Malformation was not greater than expected in offspring of rats and mice.	[67-71]
**Thymol**	Human	• Topically, it is safe.	• It is not associated with increased risks of birth defects.	[21,72-76]
		• It is toxic to mucous membranes and to kidneys, liver, and central nervous system.		
	Animal	• Topically, it is safe.	• Might cause adverse reproductive effects.	
**Sea Cucumber Extract**	Human	• Topically, it is safe	• Till date, no data.	[77-81]
	Animal	• Topically, it is safe.	• Till date, no data.	
**Aloe Barbadensis Leaf Juice**	Human	• Topically, it is safe, however, not recommended for children under age of 12 years.	• Orally, it is not recommended in pregnancy or lactation.	[82-88]
		• Orally, there are adverse effects in rare cases.		
	Animal	• Topically, it is safe.	• Teratogenic effects have been reported with high oral doses in rats.	
**Peppermint Oil**	Human	• Topically, it caused skin irritation with frequent use of oil.	• It induces menstruation and, thus, it is not recommended at high oral doses during pregnancy.	[83,89-91]
		• Orally, enteric-coated capsules were not associated with adverse reactions.	• There is insufficient evidence to determine the safety of peppermint oil during lactation.	
	Animal	• The oral LD_50_ was reported at 2490 mg/kg in mice and at 2426 mg/kg in rats.	• Orally, the LD_50_ was 2490 mg/kg in mice and 2426 mg/kg in rats.	
			• The peppermint oil was used to induce menstruation and it is not recommended at high oral doses in pregnancy.	
Boswelli, and Magnesium chloride	Human	• Topically, boswelli is associated with dermatitis.	• Orally, there is lack of evidence on safe use of boswelli during pregnancy and lactation.	[92-99]
		• Orally, boswelli is associated with gastrointestinal effects including, nausea, abdominal fullness, and epigastria.	• Similarly, magnesium chloride studies failed to demonstrate risks of birth defects to the fetus.	
		• Orally, magnesium chloride is not recommended for patients with renal impairment.		
		• Both magnesium and ilex are safe for topical use.		
	Animal	• Topically, boswelli is safe in mice, rats and monkeys.	• There is lack of evidence on safe use during pregnancy and lactation.	
		• Orally, boswelli was not associated with mortality in rat and mice.		
		• In rat and monkey, there was no change in behavior, clinical, biochemical, or pathological data when boswelli was used orally.		

### Dimethyl sulfone (Methylsulfonylmethane or MSM)

Several studies showed no adverse reactions when a cream containing dimethyl Sulfone (MSM) was applied topically on a daily basis over four weeks [11]. Treatment of interstitial cystitis with MSM revealed a low rate of adverse effects [11]. Similarly, adverse drug reactions were minimal in a study that evaluated the efficacy of MSM in reduction of symptoms associated with allergic rhinitis [12]. In this study, 15 males and 35 females between the ages of 21 and 60 received 2.6 g of MSM once daily for 30 days and a subset of subjects further received 5.2 g for additional 14 days. Although the recommended dose for dietary supplementation of MSM is 1 to 6 g/day, there are no peer-reviewed studies on its long-term use in humans. MSM was reported to cause low oral toxicity in rats (LD_50_ was greater than 17 g/kg). A dose of 2 g/kg or long-term administration (1.5 g/kg for 90 days) did not cause adverse events in rats [13]. Although some studies reported that MSM can cross the placenta resulting in higher concentrations in the fetal plasma [14], to date, there have been no human data on adverse reproductive effects. Oral administration of MSM to pregnant rats at doses of 50 to 1000 mg/kg/day during organogenesis did not induce fetal anomalies. These studies concluded that No Observed Adverse Effect Level (NOAEL) for maternal and developmental toxicity is 1 g/kg/day [15,16].

### Camphor

Poisoning cases after topical use of camphor have been reviewed for adults and children between 1990 and 2003 [17]: 1) A 15-month-old boy who crawled through spilled camphor spirits developed ataxia and generalized convulsions. 2) A 2-month-old girl developed elevated serum transaminase following application of Vicks VapoRub to her chest and neck (4.8% camphor, 3 times a day for 5 days). 3) A 25-month-old boy developed delirium, visual hallucinations, and urinary incontinence after his chest was “soaked” in more than 1 ounce of camphorated oil for 80 hours (6.4 g of camphor). 4) A 9-month-old girl with 20% body surface area burns was treated with a dressing containing 9.6% camphor for 24 hours (estimated exposure to 15 g camphor) and developed severe toxicity including convulsions. 5) A 72-year-old woman developed granulomatous hepatitis following dermal application of five containers of Vicks VapoRub Ointment. After discontinuation of the use, these problems were resolved.

Of importance, several studies have shown that camphor has a low absorption rate (less than 0.1%) from medicated patches containing 46.8 mg camphor in conjunction with 37.4 mg menthol and 74.88 mg methyl salicylate [18]. Orally, the exposure of children (ages of 14 months - 5 years old) to 0.7-1.5 g of camphor was associated with fatal symptoms [19]. Another study has examined the administration of camphor to 80 postpartum women who were injected 195 mg camphor in the first day followed by daily injections of 97 mg over 3 days. In this study, only one patient developed adverse events (nausea and vomiting) [17]. Thus, the US FDA has set a limit of 11% camphor in different products, as ingestion of larger quantities may cause adverse effects such as seizures, confusion, irritability and neuromuscular hyperactivity. The LD_50_ in mice is 1.3 g/kg.

With respect to reproductive effects, studies showed that camphor can cross the human placenta when ingested orally [20]. Topically, the frequency of birth defects was not greater than baseline among 168 pregnancies where camphor was used in the first trimester as well as among 763 women who used camphor anytime during pregnancy [21]. There was no increase in the incidence of congenital abnormalities when camphor was used at high oral doses in pregnant rats and rabbits (100–1000 mg/kg/day and 50–681 mg/kg/day) [22-24]. Although few reports of camphor poisoning are available, to date, there are no reported adverse fetal effects [20,25-27]. It has been reported that women who used a suppository containing camphor during pregnancy had infants with an increased average birth weight and gestational age [28].

### Menthol

The FDA has approved methanol as a safe chemical for external use with concentrations up to 16% [29]. Acute dermal toxicity was reported with LD_50_ of 5 g/kg in rabbits. Orally, the LD_50_ values were 2.9 g/kg and 3.1 g/kg in rat and mice, respectively. It has been reported that rats given menthol at 200 mg/kg/day for 28 days had increased liver weights and vacuolization of hepatocytes [30]. With respect to reproductive effects, there has been no study examining menthol safety in humans. There were no teratogenic effects seen in the offspring of mice, rats, hamsters, or rabbits receiving doses ranging from 1 to 106 times the accepted daily intake in humans [31].

### Wintergreen oil (Gaultheria procumbens/Methyl salicylate)

Although prolonged skin contact with methyl salicylate may cause dermatitis, methyl salicylate is generally safe in topical formulations [32]. Local necrosis occurred in a 62-year-old man after 1 day use of Bengay (18.3% methyl salicylate and 16% menthol) [33]. The Bengay was applied to the forearms and legs along with periodic heating of the area with a heat pad [33]. There was evidence of tinnitus, diplopia, shortness of breath, mixed metabolic acidosis, and respiratory alkalosis [33]. The lowest lethal oral dose of methyl salicylate was reported in women at 355 mg/kg, in men at 101 mg/kg, and in children at 228 mg/kg. Of importance, a teaspoon or less of wintergreen oil has been implicated in several deaths of children under the age of 6 years [34]. It has been reported that dermal exposure to methyl salicylate was associated with LD_50_ of 2 g/kg [32]. Both the rate and extent of absorption through the skin are dependent on the exposed area as determined in equine skin [35]. The oral LD_50_ is 887 mg/kg in rats.

With respect to reproductive effects, administration of methyl salicylate (up to 0.5 mL) in rats during organogenesis increased the rate of abnormalities, particularly of the central nervous system. This dose represents 5 times the lethal adult human dose [36]. It has been reported that pregnant hamsters treated orally or topically with 175 mg/100 g methyl salicylate exhibited central nervous system teratogenicity [37]. However, there is no conclusive evidence that salicylate is teratogenic in humans [38]. When methyl salicylate was given at doses of (200, 250, or 300 mg/kg/day), there was a reported alteration in renal pelvis and urine formation in rats [39]. However, the topical application of methyl salicylate in petroleum-based grease did not cause congenital defects when used at doses up to 6000 mg/kg/day [40]. High doses of methyl salicylate (such as 250 and 500 mg/kg/day) were reported to increase the litter size. However, these doses did not result in congenital abnormalities in mice. The lower doses (100 mg/kg/day) had no observable adverse reproductive effects [41]. Importantly, the doses in these studies were significantly higher than the adult lethal human dose on mg/kg basis. The American Academy of Pediatrics recommended the use of salicylates with caution during breastfeeding [100].

### Glucosamine sulfate

It has been shown that topical application of a cream containing 0.3% glucosamine sulfate did not cause adverse effects [43]. In large clinical trials, oral glucosamine reduced progression of knee osteoarthritis and prevented joint space narrowing [44,45]. Although glucosamine is widely used in veterinary medicine [46], no teratogenic effects were reported in mice or rabbits. In a prospective controlled study of 34 pregnant women exposed to glucosamine during the first trimester, there was no increase in risk of major malformations [47].

### Sodium chondroitin sulfate

Several studies failed to report significant adverse events with topical use of a cream containing 0.78% chondroitin sulfate [43]. The use of chondroitin in combination with glucosamine is effective in treatment of knee pain since chondroitin enhances the pain-relieving action of glucosamine [48-50]. It has been reported that chondroitin and glucosamine sulfate attenuate progression of osteoarthritis [51]. Orally, the LD_50_ was greater than 10 g/kg in mice. However, mice injected with 1 mL of 2% chondroitin on day 9, 10, or 11 of gestation exhibited an increased rate of cleft palate and tail abnormalities in the offspring [52]. To date, there has been no human study on the effects of chondroitin sulfate during pregnancy and lactation.

### Eucalyptus leaf oil (Eucalyptus globulus/1, 8-cineole)

Eucalyptus leaf oil is widely used in mouthwashes and cough suppressants. However, to date, there have been no reported deaths caused by topical use of eucalyptus oil. There was only a report of fever and seizure-like motor activity in a 2-year-old boy rubbed with eucalyptus oil [53]. A 6-year-old who was exposed to eucalyptus oil exhibited slurred speech, ataxia, and muscle weakness [54]. There were minor adverse effects in two 76-year-old patients ingesting 600 mg of eucalyptus daily for 7 days [55]. Orally, the LD_50_ was 2.5 g/kg in rats. There has been no adverse outcome in mice injected on days 6 and 15 of gestation [56]. Also, there has been no evidence of adverse reproductive effects of eucalyptus oil in humans.

### Grape seed oil (Vitis vinifera)

Procyanidin B-2, a component of Grape Seed Extract, was found to be safe for topical use based on mutagenic and ocular irritation assays [57]. The proanthocyanidin extract is regarded safe for consumption at 1.4 g/kg/day [58]. There are no safety issues identified in acute and chronic studies of oral and dermal exposure to the proanthocyanidin extract in animal studies [59]. The grape seed extract was also non-mutagenic in mice [60]. Several reports showed that the LD_50_ for dermal application is greater than 2 g/kg in rats [59]. With subcutaneous injection, the lethal dose of procyanidin B-2 was greater than 2 g/kg [57]. However, to date, there have been no human data on the reproductive effects of grape seed oil.

### Vitamin E (Alpha-Tocopherol acetate)

Vitamin E is safe for topical use in different creams aiming to protect the skin against ultraviolet rays [61]. There have been four randomized double-blinded trials involving 566 women at risk of pre-eclampsia who received high doses of vitamin E in the second and third trimesters of pregnancy. In these trials, there was no difference between women exposed to high doses vitamin E and women exposed to placebo in terms of risk for stillbirth, perinatal death, preterm birth, growth restriction, or birth weight [62-65]. Similarly, among 82 infants born to women who received high doses of vitamin E (400 mg/day), there was no increase in malformations or miscarriages [62-65]. Only one infant was born with omphalocele [66]. The frequency of malformations was not increased in the offspring of rats and mice treated with vitamin E at doses hundreds to thousands times of the human doses [67-71].

### Thymol

Thymol is considered safe in topical formulations at a concentration of 0.5% [72]. However, thymol was toxic to mucous membranes as well as to the kidneys, liver, and central nervous system [72]. The oral LD_50_ was 980 mg/kg in rats, 640 mg/kg in mice, and 880 mg/kg in guinea pigs. Thymol was not associated with increased incidence of birth defects based on 52 pregnancies exposed in the first trimester of pregnancy [21]. Although thymol was used previously as part of an abortifacient paste to induce the abortion [73-75], only one fatality was reported with the paste use [76].

### Sea cucumber extract (SCE)

It has been reported that topical exposure of gingival tissue to sea cucumber extract (SCE) over three months was safe [77]. Also, the use of SCE as an oral daily supplement for six months was not associated with any adverse effects. Although SCE has been shown to have potent anti-tumor effects *in vitro* [78-81], to date, there has been no evidence on safety of SCE during pregnancy and lactation.

### Aloe Barbadensis leaf juice

Aloe is considered safe in topical formulations since hypersensitivity reaction is relatively rare [82]. Although the aloe is used in adults, it is not recommended for children under age of 12 years [83]. The aloe is used orally to relieve constipation and adverse effects were reported only in rare cases [83]. A 56-year-old woman developed hypothyroidism after high doses of aloe taken for 11 months [83]. The thyroid function tests had normalized 16 months after discontinuation of treatment [84]. Another 73-year-old woman developed acute hepatitis after ingesting aloe powder every 2 to 3 days for 5 years [85]. The liver function tests and biopsies were consistent with findings of drug-induced acute hepatitis and completely resolved after discontinuation of aloe use [85]. Similarly, a 24-year-old male developed acute drug-induced hepatitis after 3 weeks of daily use of oral aloe extract where the clinical symptoms resolved 7 days after the aloe was discontinued [86]. Thus, aloe is not recommended orally during pregnancy and/or lactation [87]. Teratogenic effects have been reported when aloe was given at high oral doses to rats [88]. However, there is no contraindication for topical use during pregnancy and/or lactation.

### Peppermint oil

Peppermint oil is commonly used and may cause skin irritation due to the presence of menthone [89]. There are no specific therapeutic or toxic dose rages for oral use. The enteric-coated capsules of the oil have been used to treat irritable bowel syndrome at doses of 0.2 to 0.4 mL three times daily without adverse events [90,91]. Also, the average daily dose of 6 to 8 drops of the oil caused no adverse effects [83]. According to the world health organization (WHO), peppermint oil is considered a safe additive at doses up to 4 mg/kg. The oral LD_50_ is 2490 mg/kg in mice and 2426 mg/kg in rats. The peppermint oil has also been used to induce menstruation and it is not recommended at high oral doses during pregnancy [90]. Although there is insufficient evidence on safety of the oil during lactation, it has been suggested that amount of the oil in OTC products is likely to be safe for breastfed infants [90].

### Boswellia and ilex

Boswellia is frequently used in topical formulations to treat osteoarthritis. However, dermatitis was reported in 4 of 62 patients who used topical preparations containing boswellia [92,93]. The boswellia is recommended for oral use at 400 mg three times daily for arthritis and 300 mg three times daily for asthma [94,95]. The common oral adverse effects are nausea, abdominal fullness, and epigastric pain [96]. Both acute and chronic toxicity studies were conducted in mice, rats, and monkeys. In these studies there was no mortality in rats and mice that received doses up to 2 g/kg. Similarly, there were no changes in behavior, clinical, biochemical, or pathological data with oral daily use of boswellia in rats and monkeys [97]. With respect to reproductive effects, there is insufficient evidence on safety of boswellia during pregnancy and lactation. Similar to boswellia, there is insufficient scientific evidence on safety of ilex during pregnancy and lactation.

### Magnesium (Magnesium chloride)

Magnesium is commonly used in topical preparations as well as a dietary supplement in doses raging from 54 to 483 mg/day [98]. It is often used to treat hypomagnesemia intravenously as 4 g in 250 mL D5W and up to a maximum rate of 3 mL/min [99]. However, magnesium chloride is not recommended for oral ingestion in patients with renal impairment [99]. The FDA has listed magnesium chloride as pregnancy category A, which means “Adequate and well-controlled studies in pregnant women have failed to demonstrate a risk to the fetus in the first trimester of pregnancy (and there is no evidence of a risk in later trimesters)” [99].

### Synthesis of findings and conclusions

Pregnant women frequently hesitate to use systemic analgesics for treatment of pregnancy-related pain due to strong perception of teratogenic risks. Among the available topical analgesics, PTMC products are used to alleviate muscle and joint pains. Although some active ingredients in PTMC are relatively toxins when used orally at high doses, these doses are thousands of times larger than those available systemically after topical use at the recommended maximum dose (i.e. 10 g/day). Thus, this review provides evidence that, when used as indicated, PTMC is apparently safe for pregnant women and their unborn babies as well as for lactating women.

## Abbreviations

PTMC: Professional therapy MuscleCare
MSM: Methylsulfonylmethane
SCE: Sea cucumber extract
FDA: Unite state food and drug administration
OTC: Over the counter
LD_50_: Lethal dose required to kill 50% of the animals
